# Probing archaeal cell biology: exploring the use of dyes in the imaging of *Sulfolobus* cells

**DOI:** 10.3389/fmicb.2023.1233032

**Published:** 2023-09-05

**Authors:** Alice Cezanne, Baukje Hoogenberg, Buzz Baum

**Affiliations:** ^1^Cell Biology Division, MRC Laboratory of Molecular Biology, Cambridge, United Kingdom; ^2^Faculty of Science, Utrecht University, Utrecht, Netherlands

**Keywords:** archaea, fluorescent imaging, live cell imaging, hyperthermophiles, molecular probes

## Abstract

Archaea are key players in many critical ecological processes. In comparison to eukaryotes and bacteria, however, our understanding of both the cell biology and diversity of archaea remains limited. While archaea inhabit a wide range of environmental conditions, many species are extremophiles, surviving in extreme temperature, salt or pH conditions, making their cell biology hard to study. Recently, our understanding of archaeal cell biology has been advanced significantly by the advent of live cell imaging *in extremis* as well as the development of genetic tools to exogenously express fluorescent proteins in some mesophilic archaeal model systems, e.g., *Haloferax volcanii*. However, for most archaeal species, especially thermophilic species or emerging model systems without well characterized genetic tools, live cell imaging remains dependent on fluorescent chemical probes to label and track the dynamics of living cells. While a wide range of fluorescent stains and markers that label different components of the cell are available commercially, their use has usually been optimized for use in a small number of eukaryotic cell systems. Here we report the successes and failures of the application of membrane, DNA, S-layer and cytoplasm markers in live cell imaging of archaea, as well as the optimization of fixation and immunolabelling approaches. We have applied these markers to the thermoacidophilic archaeon *Sulfolobus acidocaldarius,* but expect some to work in other archaeal species. Furthermore, those procedures that failed in *S. acidocaldarius* may still prove useful for imaging archaea that grow at a more neutral pH and/or at a less extreme temperature.

## 1. Introduction

Archaea were first proposed to be a distinct domain of prokaryotic life by Carl Woese and colleagues (Woese and Fox, 1977). Since then, microbiology and phylogenetic studies have greatly furthered our understanding of the tree of life, as well as the extreme diversity of archaeal species and the wide variety environmental niches they occupy. However, the genetic tools and imaging procedures required to study the cell biology of archaea remain much less well developed than those available for studies in bacteria and eukaryotes. The difficulties are compounded by the fact that many archaea are extremophiles.

Much of the core information processing and cytoskeletal machinery present in eukaryotes appears to have an archaeal origin (Lake et al., 1984; Spang et al., 2015; Zaremba-Niedzwiedzka et al., 2017). Given their close evolutionary relationship with eukaryotes, many archaea possess simpler counterparts of the core machinery found in eukaryotes. This includes machinery involved in genome organization (Peeters et al., 2015; Mattiroli et al., 2017), DNA replication initiation and its elongation (Barry and Bell, 2006), transcription (Werner, 2007), rRNA processing (Omer et al., 2000), N-linked glycosylation (Jarrell et al., 2014), the Ubiquitin-ESCRT (Endosomal Sorting Complexes Required for Transport)-proteosomal system (Zwickl et al., 1992; Nunoura et al., 2011; Hennell James et al., 2017; Hatano et al., 2022), and the actin cytoskeleton (Akıl and Robinson, 2018; Rodrigues-Oliveira et al., 2023). Understanding the cell biology of the relatively simple archaeal counterparts of eukaryotic proteins machineries can shed new light on their origins and can reveal underlying principles that are obscured by the complexity of the machinery present in eukaryotes. While recent attention has focused on the Asgard archaea from within which eukaryotes likely emerged (Eme et al., 2023), thus far only a few members of the Asgard archaea have been successfully cultivated. Moreover, in these cases the cells are present in mixed cultures, which include syntropic partners, and must be grown under anaerobic conditions (Imachi et al., 2020; Rodrigues-Oliveira et al., 2023). As a result, most cell biology studies that aim to use archaea as simple models to study eukaryotic protein machineries have focused on the related TACK superphyla (Thaum-, Aig-, Cren-, and Korarchaeota), whose members share fewer molecular features with eukaryotes than Asgard archaea, but are far more experimentally tractable. These have proved useful model systems with which to probe the minimal components needed for cellular processes in eukaryotes, as has been done for ESCRT-III dependent cytokinesis (Lindås et al., 2008; Samson et al., 2008; Pulschen et al., 2020; Tarrason Risa et al., 2020; Hurtig et al., 2023).

While many cellular processes are shared between archaea and eukaryotes, archaea also possess unique chemical and biochemical features that are not found in other domains of life. One of the most prominent examples of this is the archaeal membrane, which is composed of unique lipid structures consisting of isoprenoid chains linked to glycerol-1-phosphate backbones by an ether linkage (Koga and Morii, 2007). In contrast, bacteria and eukaryotes share a phospholipid composition of fatty acid chains, linked by an ester linkage to glycerol-3-phosphate backbones (Peretó et al., 2004). This phenomenon is commonly termed the lipid divide, and has important implications for the emergence of bacteria and archaea as distinct domains of life as well as for eukaryogenesis (Peretó et al., 2004; Koga, 2011; Lombard et al., 2012; Villanueva et al., 2021). In addition, as many archaea are extremophiles, they possess unique strategies to survive the harsh environmental conditions in which they live: be it high salt, high temperature or low oxygen. While this presents experimental challenges, as we will discuss below, understanding the cell biology of extremophiles has a wide range of applications, including within industry (e.g., in drug and vaccine delivery) and in the search for life on other planets (Patel and Sprott, 1999; Jacquemet et al., 2009; Merino et al., 2019).

As a cell biological tool, fluorescence microscopy has enabled the characterization of many biological processes in living cells (Shimomura et al., 1962; Zimmer, 2002). However, its application in the field of archaeal cell biology is still under development (Bisson-Filho et al., 2018; Pulschen et al., 2020; Charles-Orszag et al., 2021). This in part reflects the challenges one faces when working with archaea. The solubility and stability of fluorescent probes must be tested for a range of media conditions including high salt, low pH and high temperatures. Further, a number of archaeal species are anaerobic which presents a challenge for light microscopy applications. Due to differences in membrane architecture, it must further be considered whether fluorescent probes can cross the membrane to reach cytoplasmic targets or whether membrane targeting probes designed for eukaryotes and bacteria can interact with the archaeal lipid membrane at all. As most eukaryotes are studied within a narrow range of environmental conditions (neutral pH, 22–37°C), only few fluorescent probes developed for use in eukaryotes have been tested at extreme conditions. 2-photon imaging of Laurdan, for example, has been successfully used to characterize reconstituted archaeal membranes at low pH (pH 2.68) and high temperature (up to 64°C) (Bagatolli et al., 2000), however methods for imaging intact archaeal cells as they grow and divide are still in their infancy.

**Figure 1:**
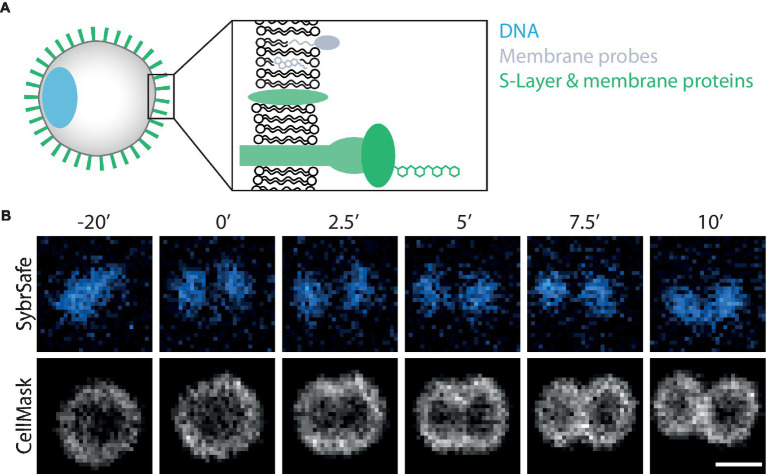
Visualizing *S. acidocaldarius*. **(A)** Schematic representation of labelling targets investigated in this study. **(B)** Time-lapse imaging of DNA and membrane dynamics in DSM 639 cells during cell division, time steps indicated in minutes above images. Cells were stained with SybrSafe and CellMask Deep Red Plasma Membrane stain and imaged at 75°C in Brock medium. Scale bar = 1 μm.

Here we explore the use of fluorescent markers for live cell imaging of *S. acidocaldarius,* currently the most experimentally tractable relative of eukaryotes. *S. acidocaldarius* is a member of the TACK superphylum, grows at pH 3 and 75°C, has a well-established molecular genetic toolbox (Lewis et al., 2021), and an ordered cell-cycle similar to that of eukaryotes (Bernander, 2007). For this analysis we explored the use of commercially available fluorescent probes to label different components of *S. acidocaldarius* including the membrane, DNA, S-layer, membrane proteins and cytoplasm (Figure 1A), as well as the optimization of different fixation methods for immunolabelling. The results of these tests will be useful to the wider archaeal community, and we hope they will help to fuel a growing interest in the cell biology of archaea (van Wolferen et al., 2022).

## 2. Materials and methods

### 2.1. Cell culturing

*S. acidocaldarius* DSM 639 (wild-type) or MW001 (uracil auxotrophic cloning strain) were grown in a shaking incubator at 75°C in Brock medium pH 2.9 supplemented with 0.1% N-Z-amine and 0.2% sucrose. MW001 cultures were supplemented with 4 μg/mL uracil. All cultures used for imaging were collected during exponential growth phase at an optical density at 600 nm (OD_600nm_) of 0.1 to ~0.4.

### 2.2. Cell labelling

All dyes in listed in Tables 1–3 were dissolved in DMSO and added to a final concentration of no greater than 0.1% (v/v). Note that much higher concentrations of DMSO (>10%) can be added to cultures without it interfering with growth. Staining was performed for either 5 min at either room temperature (23°C) or for 1 min at 75°C. The signal from dyes that stained cells over background during this time-frame did not greatly improve upon longer incubation times. Similarly, dyes that failed to label cells within 5 min at room temperature did not successfully stain cells over incubation times of up to 30 min. To settle on conditions for live imaging we tested candidate probes at different concentrations over different exposure times at room temperature. Probes that successfully stained cells at room temperature were then re-tested at the same concentrations at 75°C using different exposure times. Probes that successfully stained cells at 75°C were then tested for cytotoxicity (without illumination) by performing growth curves over 24 h in presence or absence of the dye at concentrations determined above. Only DiO(C6) was found to be cytotoxic at concentrations used for live cell imaging at either room temperature or 75°C. Note that because cells were not washing following labelling, it likely that the pool of dye present in the medium can exchange with the cellular pool – reducing the effects of photobleaching.

**Table 1:** List of membrane labels used in this study.

Probe	λ_ex_/ λ_em_ (nm)	Membrane Interaction	24°C (pH 3)		75°C (pH 3)		Toxic (75°C)
			Staining	Conc.	Staining	Conc.	
Nile Red	~552/636	Insertion into lipid core	Yes	5 μg/mL	Yes	2.5 μg/mL	No
CellMask™ Deep Red Plasma Membrane Stain	649/666	Lipophilic tail	Yes	0.5 μg/mL	Yes	1 μg/mL	No
CellMask™ Orange Plasma Membrane Stain	556/573	Lipophilic tail	Yes	0.5 μg/mL	Yes	1 μg/mL	No
Mitotracker^®^ Green FM	490/516	Insertion into lipid core	Yes	0.336 μg/mL	Diffuse cytoplasmic signal		No
Mitotracker^®^ Red CMXROS	579/599	Insertion into lipid core, Membrane potential dependent	Yes	0.265 μg/mL	Membrane and cytoplasmic signal	0.1325 μg/mL	No
BODIPY™ TR Ceramide	592/618	Insertion into lipid core	Weak signal	12.5 μg/mL	No		-
DiO C6	488/506	Either: intercalates between leaflets OR insertion or two lipid tails and fluorophore outside	Yes	2 μg/mL	No		Yes
DiO C18(3)	488/506		No		-		-
SP-DiO C18(3)	488/506		No		-		-
DiI C18(3)	550/570		No		-		-
DiI C18(3) DS	550/570		No		-		-
5,5’-Ph2-DiI C18(3)	550/570		No		-		-
SP-DiI C18(3)	550/570		No		-		-
DiA	450/585		No		-		-
DiR (DiI C18(7))	750/780		No		-		-
Mitotracker^®^ Deep Red FM	644/665	Insertion into lipid core	No		No		-
CellMask™ Green Plasma Membrane Stain	522/535	Lipophilic tail	-		No		-
Nile Blue	626/668	Insertion into lipid core	No		No		-
FM™ 4-64X	~515/640	Anchored in outer leaflets, fluorescent in hydrophobic environments	No		No		-
FM™ 1-43FX	510/626	Anchored in outer leaflets, fluorescent in hydrophobic environments	No		No		-

Names colour coded according to success of staining. Green: optimal staining; Blue: conditionally applicable staining; Grey: no staining. Hyphens indicate that the condition was not tested.

**Table 2:** List of cell contour and content markers used in this study.

Probe	Binding interaction	Readout	λ_ex_/ λ_em_ (nm)	24°C (pH 3)		24°C (pH 5)		75°C (pH 7)		EtOH Fixation Compatible	
				Staining	Staining	Staining	Conc.	Staining	Conc.		
CellTracker Green	-	Cytosol	492/517	Yes	4.65 μg/mL	-		Yes	0.93 μg/mL	-	
CellBrite	Amines	Membrane Proteins	480/513	No		Yes	1x			No	
ConA	Glycosylation	Contour (Glycosylated membrane proteins)	Multiple	No		No		No		Yes	50 μg/mL
Brilliant Blue	Amines	Contour (Membrane Proteins)	490/515	No		No		No		-	

Names colour coded according to success of staining. Green, optimal staining; Blue, conditionally applicable staining; Grey, no staining. Hyphens indicate that the condition was not tested.

**Table 3:** List of DNA labels used in this study.

Probe	λ_ex_/ λ_em_ (nm)	24°C (pH 3)		24°C (pH 7)		75°C (pH 3)		EtOH Fixation Compatible	
		Staining	Conc.	Staining	Conc.	Staining	Conc.		
SYBR™ Safe DNA Gel Stain	502/530	Yes	1:10000	Yes	1:10000	Yes	1:5000	No	
SYTO™ 11	508/527	Yes	5 μM (~2 μg/mL)	-		-		-	
SYTO™ 12	499/522	No		-		-		-	
SYTO™ 13	488/509	Weak signal		-		-		-	
SYTO™ 14	517/549	No		-		-		-	
SYTO™ 16	488/518	Yes	1 μM (~0.45 μg/mL)	-		-		-	
SYTO™ 17	621/634	No		-		-		-	
SYTO™ 21	494/517	No		-		-		-	
SYTO™ 24	490/515	No		-		-		-	
SYTO™ 59	622/645	Yes	5 μM (~2.75 μg/mL)	-		-		Yes	5 μM
SYTO™ 60	652/678	No		-		-		-	
SYTO™ 61	628/645	Yes	5 μM (~2.5 μg/mL)	-		-		-	
SYTO™ 62	652/676	Yes	5 μM (~2.75 μg/mL)	-		-		-	
SYTO™ 63	657/673	Yes	5 μM (~2.75 μg/mL)	-		-		-	
SYTO™ 64	599/619	Yes	5 μM (~2 μg/mL)	-		-		-	
GelRed^®^	279/593	No		-		-		-	
DAPI	350/470	No		No		No		Yes	10 μg/mL
Hoechst	350/461	No		No		No		Yes	1.23 μg/mL

Names colour coded according to success of staining. Green, optimal staining; Blue, conditionally applicable staining; Grey, no staining. Hyphens indicate that the condition was not tested.

### 2.3. Live cell-imaging at 75°C

Live-cell imaging was performed at 75°C using the “Sulfoscope” chamber described in Pulschen et al. (2020), with modifications to the hardware described by Hurtig et al. (2023). Briefly, 25 mm coverslips were washed with EtOH and H_2_O, then assembled into commercial Attofluor chambers (Invitrogen A7816). Chambers were filled with 300 μL of Brock medium and incubated at 75°C for at least 1 h or until the medium was dry. Afterwards, chambers were washed thoroughly with Brock medium, placed into the Sulfoscope chamber, and allowed to equilibrate to 75°C. Dyes detailed in Tables 1–3 were added to 5 mL of 75°C *S. acidocaldarius* cell culture (OD_600nm_ 0.15 to 0.3) immediately before imaging. For imaging, 400 μL of cell suspension (OD_600nm_ 0.15 to 0.3) was added into the chamber and immobilized using heated, semi-solid gelrite pads (0.6% Gelrite, 0.5× Brock medium pH 5, and a final concentration of 20 mM CaCl_2_). Pads were prepared in the following way: ~15 ml molten Gelrite Brock medium solution was added to 9cm plastic petri dishes, and allowed to set at room temperature (~5 minutes). Half-moon shapes were then cut from the plate with a 7 mm diameter circle punch, placed onto 13 mm circular coverslips, and incubated at 75°C for 5 to 10 min in a bead bath. During this period of incubation, pads equilibrated to the imaging temperature and also dried slightly, causing the edges of the pad to curve downwards. Preheated pads were then placed in the chamber onto the cell suspension, such that the concave edge of the pad was in the center of the chamber. For the cell biological analysis, cells at the border of the immobilization pad were imaged since, in this area, cells are immobilized without being subjected to mechanical stress from the overlying Gelrite. Images were acquired on a Nikon Eclipse Ti2 inverted microscope equipped with a Yokogawa SoRa scanner unit and Prime 95B sCMOS camera (Photometrics). Imaging was performed with a 60× oil immersion objective (Plan Apo 60×/1.45, Nikon) using a custom formulated immersion oil for high temperature imaging (maximum refractive index matching at 70°C, *n* = 1.515 ± 0.0005; Cargille Laboratories), using the ×2.8 magnification of the SoRa unit (equivalent to a total magnification of ×168). Images were acquired using a 15 ms exposure time and 10% laser power at intervals of 15 s for 2 to 3 h. After acquisition, XY drift was corrected using the ImageJ plugin StackReg (Thevenaz et al., 1998).

### 2.4. Imaging without fixation at room temperature

1 mL DSM 639 culture in Brock medium was allowed to cool to room temperature before staining with the dyes listed in Tables 1–3, as described above. Imaging was performed on cells confined using a 1% low melt agarose pad. In brief: a 1% low melting temperature agarose (Sigma Aldrich, A9414) was prepared in MilliQ water by microwaving until the agarose was completely dissolved. 100 μL of molten agarose solution was pipetted onto a homemade spacer slide consisting of a glass slide with 4 layers of lab tape wrapped around either end. A second glass slide was then placed on top of the agarose, which was allowed to harden for 2–5 min at room temperature. Once the agarose had hardened, the spacer slide was removed and 10 μL of labelled cell suspension was added onto the agarose pad and allowed to dry fully before a 13 mm borosilicate coverslip was placed on top for imaging. Imaging was performed using the inverted microscopy set-up described above. Images were acquired with a NIKON 100x oil immersion objective (Apo TIRF 100x/1.49) and type F2 immersion oil (Nikon) in addition to the 2.8x magnification lens in the SoRA unit giving a total magnification of 280x. Z-stack images were acquired with a 0.22 μm step size (10 slices, covering ~2 μm) using an exposure time of 50 ms with laser power set to 10% of maximum.

### 2.5. Fixation

For **Stepwise** ethanol fixation, 3 mL DSM 639 culture in Brock medium was added to 1.5 mL 4°C ethanol, incubated at 4°C for 10 min before adding a further 1.5 mL 4°C ethanol, incubated for 10 min, after which a final 4 mL of 4°C ethanol was added to a final concentration of 70%. For fixation in other buffer conditions, 3 mL of culture was spun for 3 min at 8000RPM in a table top centrifuge and resuspended in 3 mL of either Tris Buffer (25 mM Tris pH 7.4, 150 mM NaCl) or Citrate Buffer (25 mM Sodium Citrate, pH 3). For **Instant** ethanol fixation, 1 mL DSM 639 culture in Brock medium was added directly to 9 mL 77% 4°C ethanol. For **Formaldehyde** fixation 3 mL DSM 639 culture was spun for 3 min at 8000RPM in a table top centrifuge and resuspended in 1 mL 4% freshly prepared paraformaldehyde (PFA) in H_2_O and incubated at room temperature with shaking for 10 min. Cells were then washed with 1 mL phosphate-buffered saline supplemented with 0.1% Tween 20 (PBST), resuspended in phospho-buffered saline supplemented with 0.1% Triton X-100 and incubated at room temperature with shaking for 10 min in order to permeabilize. Cells were then washed with 1 mL PBST before proceeding to immunolabelling. All samples were stored at 4°C before labelling and imaging.

### 2.6. Imaging fixed cells using immunofluorescence

Immunolabelling was performed as described by Hurtig et al. (2023). Briefly, 1 mL fixed cells was spun in a tabletop centrifuge (3 min, 8000RPM) after which the supernatant was discarded and cells were washed twice in 1 mL PBST supplemented with 3% bovine serum albumin (PBSTA) to remove all remaining fixative. Cells were resuspended in a final volume of 100 μL PBSTA supplemented with 5% fetal bovine serum (FBS) and primary antibodies (in this case a lab generated anti-CdvB: Tarrason Risa et al., 2020; Hurtig et al., 2023). Cells were incubated overnight at room temperature with 500 rpm agitation before washing with 1 mL PBSTA and resuspending in a final volume of 100 μL supplemented with secondary antibodies (either AlexaFluor-488 anti-rabbit (Thermo Fisher Scientific, A11034) or AlexaFluor 546 anti-rabbit (Thermo Fisher Scientific, A11035), 1:10,000) and 50 μg/mL Concanavalin A conjugated to Alexa Fluor 647 (Thermo Fisher Scientific, C21421). Cells were incubated for 3 h at room temperature and 500 rpm agitation after which cells were washed with 1 mL PBSTA and resuspended in a final volume of 1 mL supplemented with 3 μM DAPI (4′,6-diamidino-2-phenylindole; Thermo Fisher Scientific, 62,248). For imaging, Lab-Tek chambered slides (Thermo Fisher Scientific, 177437PK) were coated with 2% polyethyleneimine (PEI) at 37°C for a minimum of 30 min. Coated chambers were washed with Milli-Q water before 200 μL cell suspension was added per well and spun down for 1 h at 750 relative centrifugal force (RCF). Imaging was performed, as for live-cell imaging, at room temperature using an exposure time of 200 ms for detection of secondary antibodies and an exposure time of 500 ms for detection of DNA. Analysis and *z*-axis maximum projections were performed using ImageJ.

## 3. Results

In this paper we report our efforts to identify dyes and conditions that can be used to image thermoacidophilic archaea live. For this analysis, a variety of probes for proteins, lipids and nucleic acids were evaluated for their capacity to stain live *S. acidocaldarius* cells at 75°C or at room temperature. Unless otherwise indicated, cells were labelled with fluorescent probes in Brock medium at pH 2.9. To ensure that cells do not move during the imaging process, cells were immobilized under a soft gel pad. For imaging at 75°C, gelrite pads (Sigma Aldrich, G1910) were placed on top of a labelled cell suspension, and cells at the edge of the pad were imaged for up to 2 h as described by Hurtig et al. (2023). For room temperature imaging, labelled cells were placed between a low melting temp agarose pad and a glass coverslip as described above, and imaged for 5–10 min. In parallel, we optimized the visualization of DNA and protein structures in fixed cells using immunofluorescence. All membrane markers tested are summarized in Table 1, S-layer and cytoplasmic markers in Table 2, and DNA markers in Table 3.

### 3.1. Imaging at 75°C

**Figure 2:**
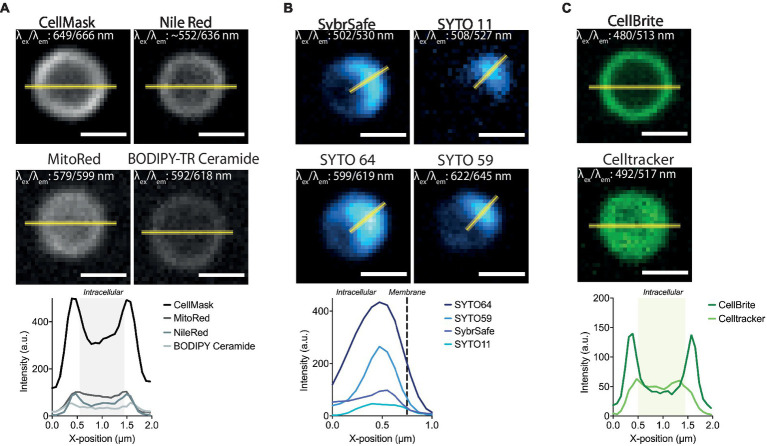
Labelling *S. acidocaldarius* at lower temperatures. **(A)** Representative membrane stains stains in Brock medium at 25°C (*top*) and intensity profiles through the equator of the cell (*bottom*). **(B)** Successful DNA stains in Brock medium at 25°C (*top*) and respective intensity profiles through the middle of the DNA signal (*bottom*). **(C)** Staining of membrane proteins (CellBrite, *left top*), general cytosolic staining (Celltracker, *right top*) stains in Brock medium at 25°C and intensity profiles through the equator of the cell (*bottom*). Line profiles are represented as a yellow line. Excitation and emission maxima for each dye are given in the top corner of each image. Scale bar = 1 μm.

The microscopy set-up used for live-cell imaging includes a heated cap and stage that functions to maintain a temperature of 75°C for several hours without dehydration (Pulschen et al., 2020). In addition, we recently added a Yokogawa SoRa unit to our confocal microscope (Azuma and Kei, 2015; Hurtig et al., 2023), enabling the resolution of discrete subcellular structures in ~1 μm diameter *S. acidocaldarius* cells. In previous live-imaging work, cells were stained with NileRed to mark the membrane and SybrSafe to label the DNA in order to visualize and characterize cell division in *S. acidocaldarius* (Pulschen et al., 2020). This is improved by using CellMask Deep Red Plasma Membrane Stain (CellMask) as a membrane stain (Figure 1B). CellMask provides a brighter and more specific membrane signal compared to NileRed, at both 75°C and room temperature (Figure 2A). Unlike NileRed, which inserts into the lipid core, CellMask is composed of a hydrophilic fluorophore attached to a lipophilic tail which inserts into the membrane. The improved resolution of the membrane signal relative to the cytoplasmic signal may therefore reflect the inability of CellMask to cross the archaeal membrane.

Importantly, under these conditions, control cells (MW001) labelled with CellMask were observed undergoing constriction over a period of ~12 min at a rate of ~0.1 μm/s (Hurtig et al., 2023), in line with speeds previously reported by Pulschen et al. (2020). This indicates that though CellMask is brighter and more specific than NileRed, it does not cause additional imaging stress. We did not observe significant phototoxicity over longer imaging periods of 2–3 h. Additional dyes that are compatible with imaging at 75°C, include the CellMask Orange Plasma Membrane Stain and Mitotracker Red CMXROS, although the latter also labels the cytoplasm (Table 1). Unfortunately, we were unable to identify a good label that could be used to image the proteinaceous surface layer at 75°C (see Table 2). A recent study however was able to visualize the cell contour of *S. acidocaldarius* by using a N-Hydroxysuccinimide (NHS)-ester functionalized Alexa Fluor dye to non-specifically label surface proteins in phospho-buffered saline at room temperature then returning cells into Brock medium for live-cell imaging at 75°C (Charles-Orszag et al., 2023).

While a range of dyes were found to strongly label the DNA of *S. acidocaldarius* cells at 75°C, the only DNA dye tested that proved compatible with long-term live cell imaging was SybrSafe (Table 3). Other dyes exhibited cytotoxicity and rapid bleaching. Nevertheless, the hardware improvements we have put in place allow for precise visualization of DNA morphology and dynamics using SybrSafe. Under these conditions, the *S. acidocaldarius* genome appears to be crescent shaped, lying close to the membrane on one side of the cell during interphase. Prior to division, it then compacts to form two foci that align with the plane of cell cleavage (Figure 1B). The organization of the genome and the membrane dynamics visualized by live cell imaging at 75°C using SybrSafe and CellMask was used to validate the other stains and probes described below.

### 3.2. Imaging at room temperature

While only a limited number of markers were found to be compatible with live imaging at 75°C, many more were found to be compatible with imaging at room temperature. In addition to the dyes described above, Mitotracker dyes, which have been used to visualize the membranes of euryarchaeota and DPANN (Maslov et al., 2018; Hamm et al., 2023), were compatible with staining the membrane of *S. acidocaldarius* cells in Brock culture medium at room temperature (Figure 2A and Table 1). Interestingly, eukaryotic type lipid stains such as BODIPY-Ceramide were also found to weakly stain the membrane of *S. acidocaldarius* cells, providing hope that other tools developed to visualize lipids in eukaryotes can be adapted to the study of the archaeal membrane. Long-chain carbocyanine dyes (e.g., DiI C18 etc), commonly used to visualize eukaryotic and bacterial membranes, did not stain the archaeal bounding membrane in culture medium or in Tris Buffer (pH 7.4). The short-chain carbocyanine DiO C6 was able to stain cells in Brock medium at room temperature, but proved to be cytotoxic at 75°C.

At room temperature, DNA could be stained with a number of STYO nucleic acid stains with different spectral properties (Figure 2B). While the signal at the start of imaging was good, these dyes suffered significant photobleaching when compared with SybrSafe. Nevertheless, these dyes could be used for labelling DNA in fixed cells, where photobleaching is less of a problem.

In addition to testing DNA and membrane dyes, we also tested a host of markers that we hoped would label membrane proteins and the S-layer in live cells. Unfortunately, these did not stain cells in low pH Brock medium, Citrate Buffer, or Tris Buffer. An exception was CellBrite, which targets primary amines. Unfortunately, CellBrite only marked the cell periphery at a high pH (Figure 2C), conditions that compromise DNA and membrane organization and, likely, *S. acidocaldarius* viability. Interestingly, the cell content marker CellTracker CMFDA (5-chloromethylfluorescein diacetate) efficiently crossed the plasma membrane to provide a uniform labeling of the *S. acidocaldarius* cytoplasm in low pH Brock medium at both room temperature and 75°C.

### 3.3. Fixation and immunofluorescence

In the absence of genetically encoded fluorescent proteins for use in hyperthermophiles, visualizing proteins and protein structures in these organisms currently relies on immunofluorescence. Unfortunately, as detergent and solvent permeabilization both greatly impact membrane integrity this precluded visualization of the membrane in fixed cells.

**Figure 3:**
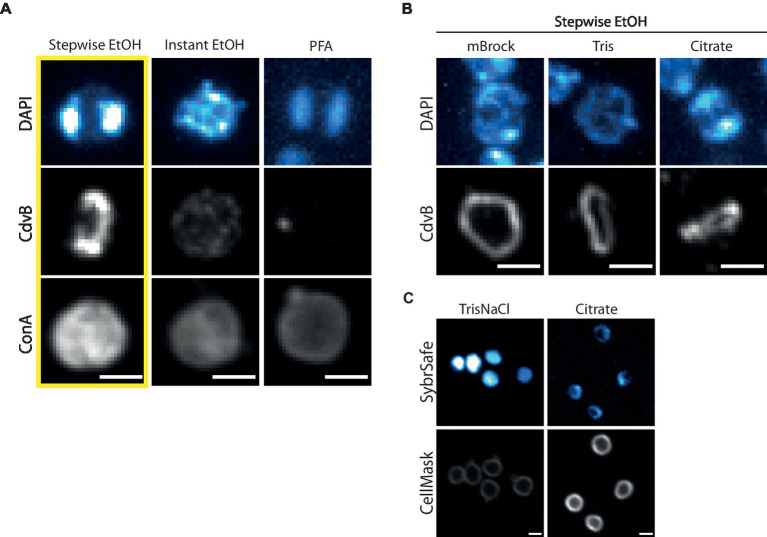
Fixation and immunofluorescence labelling of DNA, proteins and cell surface. **(A)** Comparison of different fixation methods in preserving DNA, protein and cell surface structures as represented by DAPI, immunofluorescence labelling of CdvB and ConA, respectively. Cells were fixed with Stepwise EtOH (*left*), Instant EtOH (*middle*) and Paraformaldehyde (*right*). **(B)** Stepwise Fixation performed in buffers of reduced complexity. **(C)** Live cell imaging at room temperature with SybrSafe and Cellmask in Tris (*left*) and Citrate Buffer (*right*). Scale bar = 1 μm.

We have tested and optimized a range of fixation techniques for *S. acidocaldarius*. Our standard procedure for staining *S. acidocaldarius* cells employs a “Stepwise fixation” protocol in ethanol (Figure 3A, *left*). This involves adding 3 mL of culture to 1.5 mL of ethanol. After 10 min on ice a further 1.5 mL ethanol is added and after an additional 10 min on ice, the final volume is brought to 10 mL with ethanol, to a final concentration of 70% ethanol (Bernander and Poplawski, 1997; Han et al., 2017; Zhang et al., 2019). Importantly, this fixation protocol yields a DNA signal similar to that observed by live cell imaging. Cells fixed in this way, can also be effectively labelled with antibodies targeting ESCRT-III homologues to reveal division rings like those reported in previous studies (Samson et al., 2008, 2011; Tarrason Risa et al., 2020; Hurtig et al., 2023). In addition, we are able to use fluorescently conjugated Concanavalin A (ConA) to label glycosylated proteins and the cell contour in ethanol fixed cells. While this stain has been used as a proxy for the S-layer, which we know to be heavily glycosylated in *S. acidocaldarius* (Peyfoon et al., 2010), ConA likely also marks a range of other glycosylated membrane proteins. Note that ConA also causes cells to aggregate in a concentration dependent manner.

We attempted to streamline the two-step ethanol fixation protocol by adding 1 mL of culture directly to 9 mL of 77% ethanol, yielding a final concentration of 70% ethanol. Unfortunately, this “Instant fixation protocol” failed to preserve the structure of neither DNA nor division rings (Figure 3A, *middle*). While attempts to fix cells with formaldehyde (either paraformaldehyde, glutaraldehyde or a combination of the two) were able to fix DNA so that its organization resembled that seen during live cell imaging, it did not preserve division rings (Figure 3A, *right*). Formaldehyde fixation was able to preserve ConA labelling of the membrane/S-layer, however, the staining appeared weaker than when using the Stepwise ethanol fixation. Combining formaldehyde fixation with ethanol fixation by replacing the detergent permeabilization portion of the formaldehyde fixation protocol with the stepwise ethanol fixation protocol did not appear to preserve CdvB division ring structures (data not shown).

As the Stepwise ethanol fixation was the only method tested that faithfully preserved DNA organization and division rings, we used this as a starting point to test the impact of different buffer conditions on immunolabelling (Figure 3B). For this analysis, cells were spun down and resuspended in either a minimal Brock medium (mBrock: pH 5, no supplementation with NZ-amine or FeSO4), Tris or Citrate buffer, before being fixed using the Stepwise ethanol protocol described above. Interestingly, in both mBrock and Tris Buffer, CdvB protein structures appeared smoother and more continuous than when fixation was performed in Brock culture medium. However, DNA organization was entirely disrupted in Tris Buffer, in both fixed and live cell imaging at room temperature (Figure 3C). By contrast, fixation in Citrate buffer preserved both DNA and protein organization, but the signal was reduced in both cases compared to that observed following fixation in Brock medium. While the ESCRT-III signal appeared slightly more uniform in Citrate buffer, until the advent of thermostable GFP variants that work in *Sulfolobus,* it will not be possible to determine whether or not this represents the ring in its physiological state.

## 4. Discussion and conclusion

Here we present a set of tools and protocols that can be used to image live and fixed *S. acidocaldarius* cells. By cataloguing both the successes and failures of different visualization methods and dyes, we hope this paper will provide the archaeal biology field with a starting point from which to optimize the imaging of a range of archaea. Similar studies have been conducted for bacterial species which lack an established genetic toolbox (Atwal et al., 2016). Importantly, this analysis reveals that archaeal membranes can be labelled with a variety of lipid probe architectures – including probes that insert into the core of the lipid membrane, probes that intercalate between lipid tails, probes with a lipophilic anchor, and probes that are sensitive to membrane properties. This suggests that it may be possible to adapt other techniques used to visualize and characterize the membrane in eukaryotes for use in archaea, e.g., to localize specific lipid domains or species (Höglinger et al., 2017) or to measure membrane properties and organization (Klymchenko and Kreder, 2014; Colom et al., 2018). As the archaeal membrane is chemically, structurally, and functionally distinct from bacterial or eukaryotic membranes, this type of biophysical characterization will be an interesting direction for future work.

A number of the membrane dyes tested here are compatible with aldehyde fixation and can be used to complement immunofluorescence investigations in species that are amenable to aldehyde fixation. Unfortunately, however, *S. acidocaldarius* cells did not respond well to aldehyde fixation, even when used in conjunction with EtOH fixations. While fixation via the stepwise addition of ice cold EtOH preserves cell shape and DNA morphologies, this likely compromises the membrane. Thus, it will be important to try other methods, e.g., cryofixation, to visualize the membrane in fixed cells. We also noted that the medium in which the cells are fixed has a significant impact on the quality of fixation, especially in regards to DNA morphology.

While we have optimized our protocols for imaging *S. acidocaldarius* cells, it is hoped that these protocols can be adapted to label other archaeal species, as well as other thermoacidophilic organisms. By sharing this information, we hope to assist in the further development of a transparent and collaborative archaeal research community.

## Data availability statement

The original contributions presented in the study are included in the article/supplementary material, further inquiries can be directed to the corresponding author.

## Author contributions

AC and BB conceived the study with input from BH. Live cell imaging (RT and 75°C) was performed by AC, immunofluorescence and fixed cell imaging was performed by BH. AC prepared the figures and text with input from BH and BB. All authors contributed to the article and approved the submitted version.

## Funding

AC was funded by an EMBO Postdoctoral fellowship (ALTF_1041-2021) and a Marie Sklodowska-Curie Individual Fellowship (101068523) provided by UKRI. BH was supported by Wellcome Trust (203276/A/16/Z). BB received support from the MRC LMB, the Wellcome Trust (203276/Z/16/Z) and (222460/Z/21/Z), the VW Foundation (94933), the Life Sciences–Moore-Simons Foundation (735929LPI), and from the Gordon and Betty Moore Foundation’s Symbiosis in Aquatic Systems Initiative (9346).

## Conflict of interest

The authors declare that the research was conducted in the absence of any commercial or financial relationships that could be construed as a potential conflict of interest.

## Publisher’s note

All claims expressed in this article are solely those of the authors and do not necessarily represent those of their affiliated organizations, or those of the publisher, the editors and the reviewers. Any product that may be evaluated in this article, or claim that may be made by its manufacturer, is not guaranteed or endorsed by the publisher.
